# Leukocyte Production of Inflammatory Mediators Is Inhibited by the Antioxidants Phloretin, Silymarin, Hesperetin, and Resveratrol

**DOI:** 10.1155/2014/938712

**Published:** 2014-02-24

**Authors:** Jezrom B. Fordham, Afsar Raza Naqvi, Salvador Nares

**Affiliations:** Department of Periodontics (MC 859), College of Dentistry, University of Illinois at Chicago, 801 South Paulina Street, 458 DENT, Chicago, IL 60612-7212, USA

## Abstract

Antioxidants possess significant therapeutic potential for the treatment of inflammatory disorders. One such disorder is periodontitis characterised by an antimicrobial immune response, inflammation, and irreversible changes to the supporting structures of the teeth. Recognition of conserved pathogen-associated molecular patterns is a crucial component of innate immunity to Gram-negative bacteria such as *Escherichia coli*, as well as the periodontal pathogen *Aggregatibacter actinomycetemcomitans*. In this study, we investigated the antioxidants Phloretin, Silymarin, Hesperetin, and Resveratrol to ascertain whether they altered the production of inflammatory mediators by innately-activated leukocytes. Peripheral blood mononuclear cells were stimulated with lipopolysaccharide purified from *Aggregatibacter actinomycetemcomitans*, and the production of cytokines, chemokines, and differentiation factors was assayed by enzyme-linked immunosorbent assay, cytometric bead array, and RT-PCR. Significant inhibition of these factors was achieved upon treatment with Phloretin, Silymarin, Hesperetin, and Resveratrol. These data further characterise the potent anti-inflammatory properties of antioxidants. Their ability to inhibit the production of inflammatory cytokines, chemokines, and differentiation factors by a heterogeneous population of leukocytes has clear implications for their therapeutic potential *in vivo*.

## 1. Introduction

Circulating peripheral blood mononuclear cells (PBMCs) comprise a heterogeneous population of leukocytes including cells from the lymphoid system (predominantly T-cells, B-cells, and NK cells) and myeloid system (principally monocytes). These cells express a range of pathogen-recognition receptors (PRRs) which recognise highly conserved pathogen-associated molecular patterns (PAMPs) expressed by bacteria, viruses, fungi, mycoplasma, and parasitic protozoa [1]. The largest family of PRRs are the Toll-like receptors (TLRs), the canonical member being TLR4 which recognises the lipopolysaccharide (LPS) present in the cell wall of Gram-negative bacteria, including *Escherichia coli (E. coli)* and *Aggregatibacter actinomycetemcomitans (Aa)* [2, 3]. TLR4 expression within the PBMC population is mainly by the myeloid-derived cells, being monocytes, macrophages (MΦs), and neutrophils [4, 5]. TLR4 ligation induces a program of gene expression leading to coordinated changes in surface molecule expression and the release of soluble mediators that promote immune surveillance, antigen presentation, and local inflammation [6].

Antioxidants (AOs) are present in a variety of plants (including fruits, berries, and nuts) and promote health by removing damaging free-radicals from the cellular environment, inhibiting the production of inflammatory mediators, and blocking carcinogenic processes. The therapeutic application of a number of AOs in disease is currently under investigation. Phloretin, present at high levels in fruits, such as apples, pears, and strawberries, can exert anti-inflammatory and immunosuppressive effects on both lymphoid- and myeloid-derived cells. Phloretin prevents T cell proliferation by mechanisms that include the inhibition of CD69 and CD25 expression (two important accessory signals during T cell activation) [7]. Phloretin also suppresses many of the proinflammatory and cytotoxic functions of innately activated MΦs, key orchestrators of inflammation, and central mediators of immunopathology [8]. Another flavanoid Hesperetin, present in many citrus fruits, has demonstrated a wide range of pharmacological effects that include the inhibition of inflammatory responses [9]. The milk thistle extract Silymarin is a complex of 7 flavonolignans and polyphenols, which have been shown to inhibit LPS-induced cytotoxicity in several settings, including LPS-induced neurotoxicity via suppression of microglial activation [10].

Other compounds, such as the phytoalexin Resveratrol, possess similar properties. Resveratrol is found in grapes and wine, and recent evidence has shown that it possesses several anti-inflammatory and immunosuppressive properties [11]. Many of the beneficial effects of Resveratrol, such as its cancer-preventing potential, are the result of its inhibitory effect on NF-*κ*B activation [12].

The aims of this study were to further characterize the anti-inflammatory potential of these AOs. To this end, we utilised PBMCs as a population of responder cells, constituting the majority of long-lived infiltrating leukocytes present during the inflammatory response.

## 2. Material and Methods

### 2.1. Cell Culture

Whole blood, including PBMCs, was obtained from healthy donors (*n* = 3, Sylvan N. Goldman Oklahoma Blood Institute, Oklahoma City, OK, USA) by density gradient centrifugation (Ficoll-Paque plus, GE Healthcare, Piscataway, NJ, USA) as previously described [13]. Silymarin (purity ≥ 95%), Hesperetin (purity ≥ 95%), Resveratrol (purity ≥ 99%), and Phloretin (purity ≥ 99%) were purchased from Sigma-Aldrich (St Louis, MO, USA). Dimethyl sulfoxide (DMSO) (purity = 99.90%) was purchased from EMD Millipore (Billerica, MA, USA). Silymarin, Hesperetin, Resveratrol, and Phloretin were prepared as 0.1 M stock solutions in DMSO. Combinations of AOs had a final combined concentration of 0.1 M.

PBMCs were seeded at either 4 × 10^5^ or 1.2 × 10^6^ cells in 96- or 48-well tissue culture plates (BD Biosciences, Franklin Lakes, NJ, USA), respectively. PBMCs were pretreated with single AOs at a concentration of 1 × 10^−4^ M or combinations of AOs with a combined concentration of 1 × 10^−4^ M for 2 hrs prior to stimulation. PBMCs were then stimulated for 16 hrs with purified LPS isolated from the bacterium Aggregatibacter *actinomycetemcomitans*, strain Y4 (Aa Y4), at a concentration of 1 *μ*g/mL in the presence of AOs. Media were collected and spun at 1600 RPM, and cell-free supernatants were transferred to fresh tubes and stored at −20°C. Cell viability was assayed using CellTiter 96 AQueous One Solution Reagent (Promega, Madison, WI, USA) following the manufacturer's guidelines. 20 *μ*L of the reagent was added to each well and incubated for 2 hrs under culture conditions. After 2 hrs, the absorbance at 490 nm was recorded using a 96-well plate reader.

Our previous studies using primary MΦs and our preliminary experiments utilising whole blood confirmed significant human tumour necrosis factor-alpha (TNF-*α*) production in response to Aa Y4 LPS [14]. To establish the optimal concentrations of AOs and LPS for this study, we performed dose and kinetic experiments. The doses of the AOs examined were 1 × 10^−4^, 1 × 10^−5^, and 1 × 10^−6^ M while the challenge concentrations of Aa Y4 LPS were 1, 10, 100, and 1000 ng/mL. Following a protocol, previously published by Thurm et al. [13], whole blood was stimulated with several concentrations of *E. coli* LPS or Aa Y4 LPS and TNF-*α* production quantified by ELISA over 3 time-points: 16, 24, and 48 hrs. From these data, the concentration of 1 *μ*g/mL of Aa Y4 LPS and a time-point of 18 hrs were chosen as they resulted in a final concentration of TNF-*α* being at the top end of the quantitative range of the ELISA. For the AOs, a concentration of 1 × 10^−4^ M (equating to 0.01% DMSO) and a preincubation period of 2 hrs were found to be the optimal culture conditions that did not adversely affect cell viability.

### 2.2. ELISA and Multiplex Assays

TNF-*α* capture and detection Abs were purchased from Life Technologies (Carlsbad, CA, USA) and used for the development of a TNF-*α* sandwich ELISA according to the manufacturer's instructions. Cell culture supernatants were analyzed using a 96-well plate reader with absorbance at 450 nm (reference 650 nm). Human Cytokine Fluorokine MAP kit was purchased from R & D Systems (Minneapolis, MN, USA) and assays were performed according to manufacturer's instructions. Readouts included TNF-*α*, interleukin- (IL-) 1*α* (IL-1*α*), IL-1*β*, IL-1Ra, IL-2, IL-4, IL-5, IL-6, CXCL8/IL-8 IL-10, IL-17, CCL3 (macrophage inflammatory protein-1*α* (MIP-1*α*)), CCL4 (MIP-1*β*), interferon-*γ* (IFN-*γ*), granulocyte-colony stimulating factor (G-CSF), granulocyte/macrophage-colony stimulating factor (GM-CSF), CCL2 (macrophage chemotactic protein-1 (MCP-1)), CCL5 (regulated on activation, normal T cell expressed and secreted (RANTES)), CXCL5 (epithelial cell-derived neutrophil-activating protein-78 (ENA-78)), vascular endothelial growth factor (VEGF), thrombopoietin (TPO), and fibroblast growth factor basic (FGFb). Supernatants were assayed on a Bio-Plex 200 System (Bio-Rad, Hercules, CA, USA).

### 2.3. RT-PCR Assays

Total RNA was extracted from homogenized cells using the miRNeasy kit (Qiagen, Valencia, CA, USA) according to the manufacturer's protocol. RNA was reverse transcribed using SSRT-III first strand cDNA synthesis kit (Invitrogen, Carlsbad, CA, USA). Real-time PCR was performed with Fast Plus EvaGreen qPCR Master Mix (Biotium Inc, Hayward, CA, USA) to detect transcript levels of TNF-*α* and I*κ*B-*α*. Primer sequences (Sigma-Aldrich) for TNF-*α* were Forward: CCCTTTATTACCCCCTCCTTCA, Reverse: ACTGTGCAGGCCACACATTC, and for I*κ*B-*α* they were Forward: ATCAGCCCTCATTTTGTTGC, Reverse: ACCACTGGGGTCAGTCACTC.

### 2.4. Data Analysis

Statistics were performed using Student's two-tailed *t*-test or one-way analysis of variance (ANOVA) (GraphPad Prism, GraphPad Software, La Jolla, CA, USA). Statistical significance is indicated by an asterisk ∗, where *P* < 0.05. Error bars on all graphs indicate standard error of mean (SEM) or standard deviation (SD).

## 3. Results

### 3.1. Stimulation of Whole Blood and PBMCs with Aa Y4 LPS

**Figure 1:**
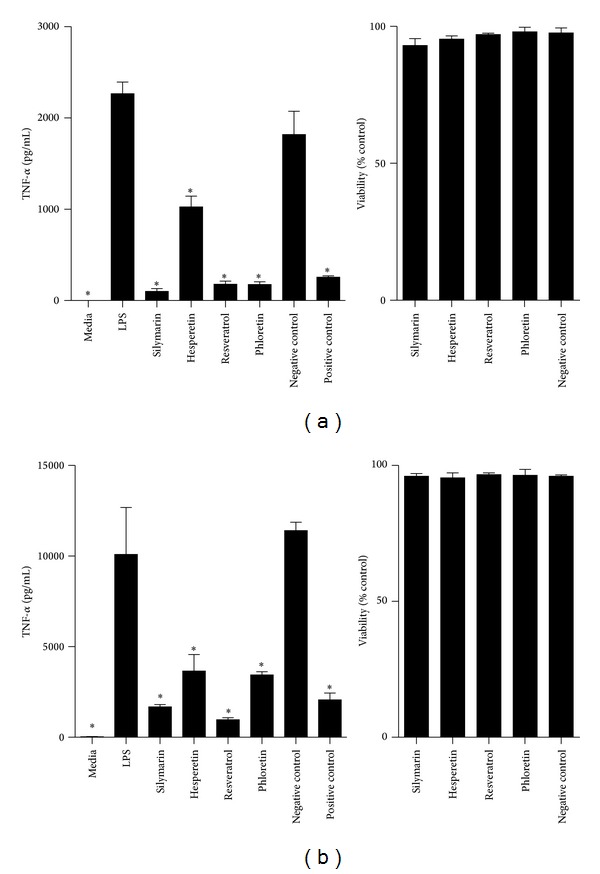
Stimulation of whole blood and PBMCs with Aa Y4 LPS. Human whole blood (a) and PBMCs (b) were cultured for 18 hrs in media alone, or treated with Phloretin, Silymarin, Hesperetin, or Resveratrol at a concentration of 1 × 10^−4^  M for 2 hrs prior to Aa Y4 LPS (1 *μ*g/mL) challenge. Negative control = LPS stimulation with 0.01% DMSO. Positive control = LPS stimulation with prednisone (10 *μ*M) preincubation. After 18 hrs, cell viability was assayed, and the concentration of TNF-*α* in the culture supernatant was measured by ELISA. Mean and SD of 3 biological replicates are displayed. Student's two-tailed *t*-test was applied, *P < 0.05 indicating statistical significance versus LPS.

Our previous studies using primary MΦs and our preliminary experiments utilising whole blood confirmed significant production of TNF-*α* in response to Aa Y4 LPS [14]. To establish the optimal concentrations of AOs and LPS for this study, we performed dose and kinetic experiments. The doses of the AOs examined were 1 × 10^−4^, 1 × 10^−5^, and 1 × 10^−6^ M while the challenge concentrations of Aa Y4 LPS were 1, 10, 100, or 1000 ng/mL. Whole blood was stimulated with several concentrations of *E. coli* or Aa Y4 LPS and TNF-*α* production quantified by ELISA over 3 time-points: 16, 24, and 48 hrs. From these data the concentration of 1 *μ*g/mL of Aa Y4 LPS and a time-point of 18 hrs were chosen as they resulted in a final concentration of TNF-*α* being at the top end of the quantitative range of the ELISA. For AOs, a concentration of 1 × 10^−4^ M (equating to 0.01% DMSO) and a preincubation period of 2 hrs was selected. Whole blood or PBMCs were cultured in media alone, media containing 0.01% DMSO, stimulated with 1 *μ*g/mL of Aa Y4 LPS, or LPS in the presence of DMSO for 18 hrs. Subsequently TNF-*α* production and cell viability were assayed (Figures 1(a) and 1(b)). Unstimulated whole blood and PBMCs did not produce detectable levels of TNF-*α* while the addition of LPS resulted in significant TNF-*α* production. The presence of DMSO alongside LPS did not impact upon the production of TNF-*α*, discounting the possibility that any effect the AOs may have would be due to the carrier. Similarly, cell viability was not significantly altered following the addition of LPS, 0.01% DMSO, or LPS + 0.01% DMSO thus indicating that any alteration in the cytokine profile with the addition of AOs was not an indirect effect of decreased responder cell viability.

### 3.2. Cytokine Profile of PBMCs Activated with Aa Y4 LPS

**Figure 2:**
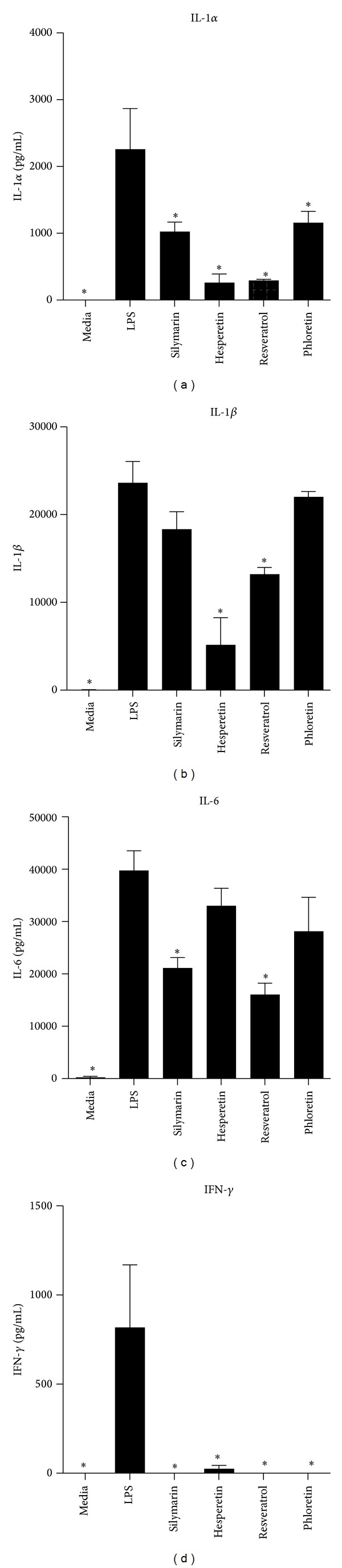
Production of the proinflammatory cytokines IL-1*α*, IL-*β*, IL-6, and IFN-*γ*. Human PBMCs were cultured for 18 hrs in media alone, stimulated with Aa Y4 LPS (1 *μ*g/mL), or treated with Phloretin, Silymarin, Hesperetin, or Resveratrol, at a concentration of 1 × 10^−4^ M for 2 hrs prior to LPS challenge. After 18 hrs, supernatant levels of (a) IL-1*α*, (b) IL-1*β*, (c) IL-6, and (d) IFN-*γ* were assayed by cytometric bead array. ANOVA was performed for statistical analysis, *P < 0.05 indicating statistical significance versus LPS, *n* = 3 independent donors.

4 × 10^5^ PBMCs were cultured in media alone or stimulated with LPS for 18 hrs and the supernatant was assayed for the cytokines and chemokines (Table 1, supplementary data, available online at http://dx.doi.org/10.1155/2014/938712). Upon stimulation with LPS, significant increases were observed in TNF-*α*, IL-1*α*, IL-1*β*, G-CSF, GM-CSF, IFN-*γ*, IL-6, IL-10, MCP-1, CCL5, MIP-1*α*, MIP-1*β*, and IL-Ra production. No significant increase was observed in CXCL5, IL-8, or VEGF levels, while the cytokines IL-4, FGF basic, IL-2, IL-5, IL-17, and TPO were undetectable. No significant decrease in cell viability was observed with LPS stimulation (Figure 2(a)).

### 3.3. Treatment with the Phloretin, Silymarin, Hesperetin, and Resveratrol Inhibits the LPS Response

**Figure 3:**
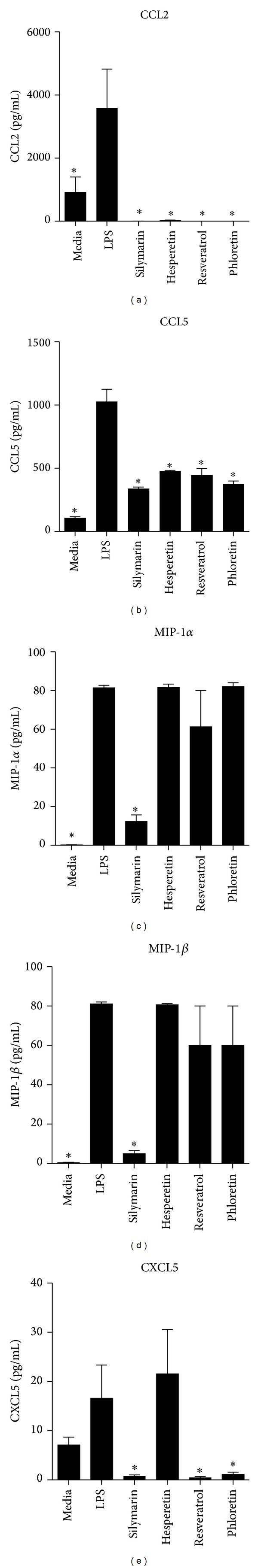
Production of the chemokines CCL2, CCL5, MIP-1*α*, MIP-1*β*, and CXCL5. Human PBMCs were cultured for 18 hrs in media alone, stimulated with Aa Y4 LPS (1 *μ*g/mL), or treated with Phloretin, Silymarin, Hesperetin, or Resveratrol at a concentration of 1 × 10^−4^ M for 2 hrs prior to, and throughout, stimulation. After 18 hrs, the concentrations of (a) CCL2, (b) CCL5, (c) MIP-1*α*, (d) MIP-1*β*, and (e) CXCL5, were assayed by cytometric bead array. ANOVA was performed for statistical analysis, *P < 0.05 indicating statistical significance versus LPS. ^#^Outside of range (above), *n* = 3 independent donors.

**Figure 4:**
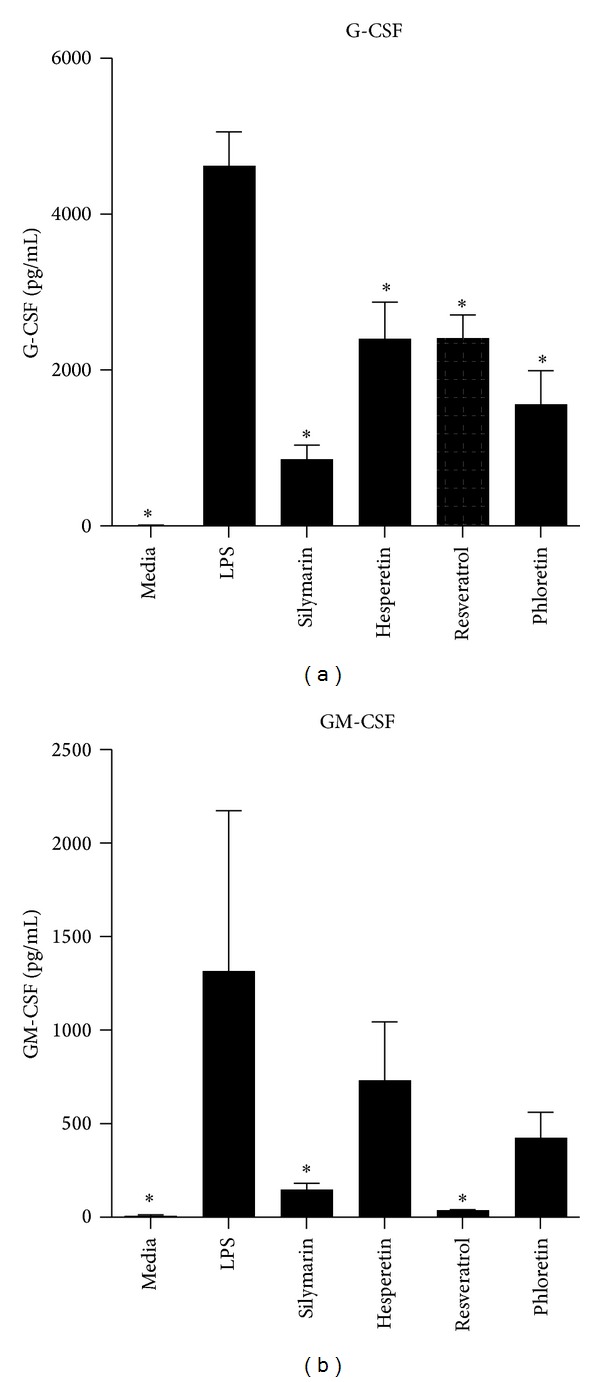
Production of the differentiation factors G-CSF and GM-CSF. Human PBMCs were cultured for 18 hrs in media alone, stimulated with Aa Y4 LPS (1 *μ*g/mL), or treated with the Phloretin, Silymarin, Hesperetin, or Resveratrol at a concentration of 1 × 10^−4^ M for 2 hrs prior to LPS challenge. After 18 hrs, the concentrations of (a) G-CSF and (b) GM-CSF were assayed by cytometric bead array. ANOVA was performed for statistical analysis, *P<0.05 indicating statistical significance versus LPS, *n* = 3 independent donors.

**Figure 5:**
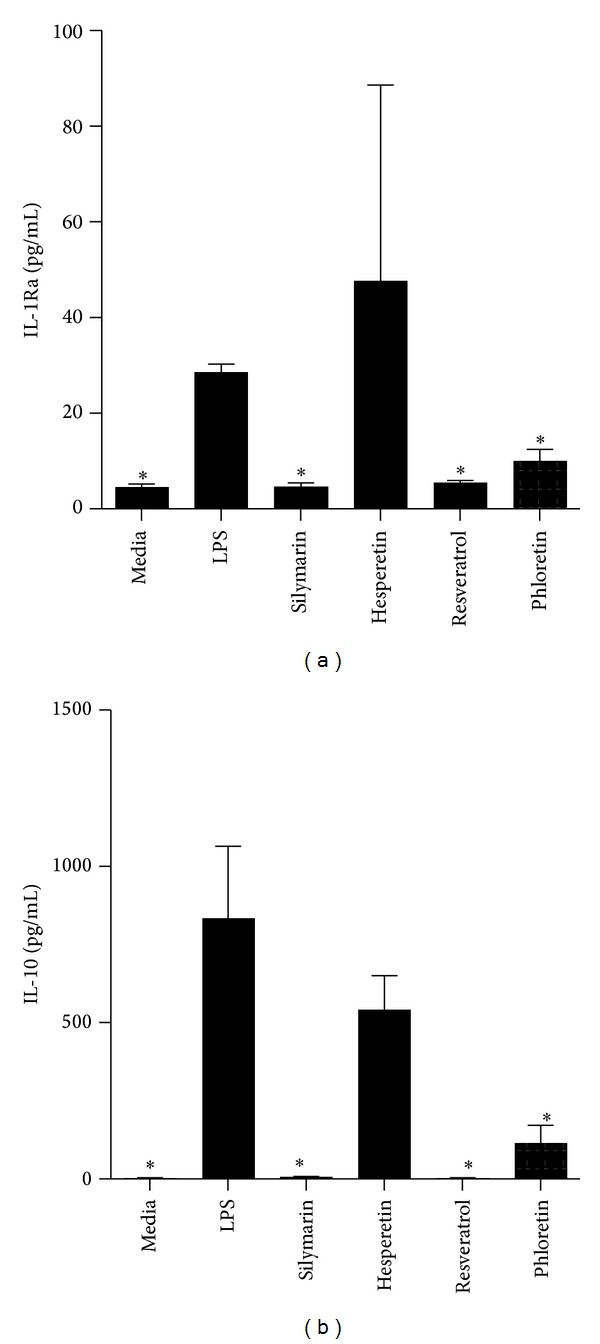
Production of the anti-inflammatory factors IL-1Ra and IL-10. Human PBMCs were cultured for 18 hrs in media alone, stimulated with Aa Y4 LPS (1 *μ*g/mL), or treated with Phloretin, Silymarin, Hesperetin, or Resveratrol at a concentration of 1 × 10^−4^ M for 2 hrs prior to LPS challenge. After 18 hrs, the concentrations of (a) IL-1Ra and (b) IL-10 were assayed by cytometric bead array. ANOVA was performed for statistical analysis, *P < 0.05 indicating statistical significance versus LPS, *n* = 3 independent donors.

PBMCs were pretreated with AOs at a concentration of 1 × 10^−4^ M for 2 hrs prior to stimulation with LPS for an additional 18 hrs. Phloretin, Silymarin, Hesperetin, and Resveratrol inhibited the production of IL-1*α*, while Hesperetin and Resveratrol decreased levels of IL-1*β* (Figures 2(a) and 2(b)). Phloretin, Silymarin, Hesperetin, and Resveratrol also inhibited the production IL-6: a cytokine which functions alongside TNF-*α* and IL-1*α*/*β* in many aspects of innate immunity (Figure 2(c)). These AOs also abolished the small amount of IFN-*γ* induced by LPS stimulation (Figure 2(d)). The release of several chemokines was inhibited by AO treatment. CCL2 is the principle chemokine for monocytes, and its production was virtually abolished by the addition of any one of the six AOs tested (Figure 3(a)). The AOs also inhibited the production of another chemokine for monocytes, CCL5 (Figure 3(b)). The structurally similar chemokines MIP-1*α* and MIP-1*β* were both inhibited by Silymarin but not by any other AO (Figures 3(c) and 3(d)). Silymarin, Phloretin, and Resveratrol had a similar effect on CXCL5, a chemokine which stimulates neutrophil chemotaxis (Figure 3(e)). Neither of the AOs significantly reduced levels of IL-8 (data not shown). In addition to the suppression of inflammatory mediators and chemokines, a reduction in the differentiation factors G-CSF and GM-CSF was also observed. Phloretin, Hesperetin, Silymarin, and Resveratrol inhibited G-CSF production (Figure 4(a)), while only Silymarin and Resveratrol had a similar effect on GM-CSF (Figure 4(b)). Interestingly three of these AOs, Phloretin, Silymarin, and Resveratrol, also inhibited the production of the anti-inflammatory cytokine IL-1Ra, a soluble decoy receptor for IL-1, and the anti-inflammatory and immunosuppressive cytokine IL-10 (Figures 5(a) and 5(b)).

### 3.4. Inhibition of TNF-*α* Is Present at the Level of mRNA Transcription and NF-*κ*B Activation

**Figure 6:**
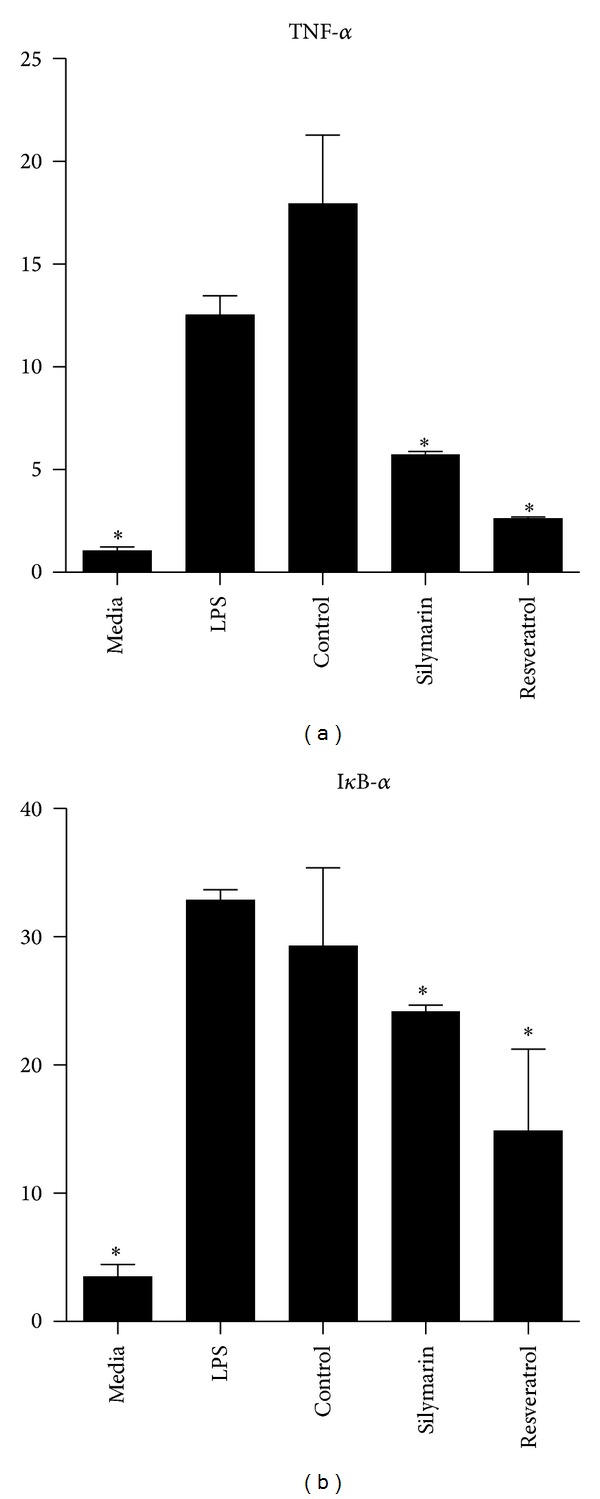
Expression of TNF-*α* and I*κ*B-*α* mRNA. PBMCs were cultured for 4 hrs in media alone, stimulated with Aa Y4 LPS (1 *μ*g/mL), or treated with Silymarin or Resveratrol at a concentration of 1 × 10^−4^ M for 2 hrs prior to LPS challenge. PBMCs stimulated with LPS +/1 0.01% DMSO constituted the control. After 4 hrs, (a) TNF-*α* and (b) I*κ*B-*α* mRNA expression levels were assayed by RT-PCR. Data is mean and SD of 3 technical replicates, 1 biological donor. Results were confirmed using a second biological donor. Student's two-tailed *t*-test was applied, *P < 0.05 indicating statistical significance versus LPS.

Next, we proceeded to investigate whether the reduced production of inflammatory mediators was due to decreased mRNA transcription. PBMCs were challenged with LPS, in the presence or absence of the highly inhibitory AOs Silymarin or Resveratrol. After 4 hrs, the cells were harvested and TNF-*α* mRNA levels were assayed by RT-PCR. Compared to LPS controls, TNF-*α* mRNA expression levels were reduced by greater than 2-fold upon treatment with Silymarin or Resveratrol (Figure 6(a)). To further investigate the inhibitory mechanism present,we performed RT-PCR for I*κ*B-*α* expression, a factor immediately downstream NF-*κ*B signaling. LPS treatment of PBMCs resulted in a 30-fold increase in I*κ*B-*α* mRNA expression compared to untreated PBMCs (Figure 6(b)). Treatment with the AOs Silymarin and Resveratrol resulted in reduced I*κ*B-*α* expression (approximately 25 and 50% reduction, resp.).

## 4. Discussion

Inflammation is a critical initial response to physical injury or to the plethora of human pathogens. However, the occurrence of exaggerated, prolonged, or inappropriate inflammatory responses can be equally deleterious to the host and if unchecked can cause significant tissue damage. Several considerations must be made when evaluating novel anti-inflammatory agents for therapeutic purposes, most importantly, its effectiveness (i.e., number of proinflammatory components targeted and the magnitude of inhibition) and pharmacological properties including sufficient bioavailability and the absence of toxicity. In the case of AOs, cytotoxicity at active concentrations does not appear to be a significant problem, nor stability, as previous studies, and our own data, have shown [14–16]. Bioavailability of physiologically active concentrations, particularly plasma levels, has been more of an issue [17, 18]. While it has proven difficult to achieve plasma concentrations of greater than 1 *μ*M for these AOs we primarily envision their use as topical treatments in which far higher concentrations have been achieved [19]. Together, our data show that the anti-inflammatory properties of naturally occurring AOs including Phloretin, Silymarin, Hesperetin, and Resveratrol encompass a wide range of inflammatory mediators including key proinflammatory cytokines, chemokines, and differentiation factors. Importantly these AOs did not impact cell viability at concentrations capable of exerting a profound inhibition of the proinflammatory response to LPS, one of the most potent inducers of immune activation and inflammation. We propose that these AOs may be of therapeutic benefit when used as topical treatments.

Most strikingly, these AOs displayed the ability to reduce the production of the key proinflammatory cytokines, TNF-*α*, IL-1*α*, IL-1*β*, IL-6, and IFN-*γ*. However, we were unable to confirm any inhibitory effects of AOs on IL-8/CCL8. This may be due to donor and/or technical variability. Further study is required to confirm any effects of AOs on IL-8 production by PBMCs and potential reduction in neutrophil chemotaxis. The central importance of TNF-*α* in the inflammatory process has been highlighted by the highly successful introduction of anti-TNF-*α* therapies for the treatment of a variety of inflammatory disorders [20–25]. Moreover, IL-1 can act synergistically with TNF-*α* for the local and systemic induction of numerous inflammatory genes [26]. TNF-*α* inhibition has been achieved via the use of monoclonal Abs, receptor fusion proteins, or small molecular inhibitors of the TNF-*α* signaling pathway [21, 22]. Although anti-TNF-*α* based therapy has been clinically successful, several notable caveats including an elevated risk of opportunistic infection, the induction of auto-Abs, injection-site and infusion reactions, and the exacerbation of previously quiescent conditions (such as multiple sclerosis or aplastic anemia) have been reported [24].

Production of the pluripotent cytokine IL-6 was also decreased by treatment with the same AOs. IL-6 is a key mediator of the acute phase of inflammation and is produced by monocytes and MΦs in response to innate stimuli such as LPS or the proinflammatory cytokines TNF-*α* and IL-1 [27, 28]. IL-6 is also important in the transition from the acute phase of inflammation in which neutrophils are the main component of immune infiltrate to the later stage in which monocytes are the predominant cell type being recruited [29]. The suppression of IL-6 production in addition to TNF-*α* and IL-1 may further inhibit the inflammatory response. The observation that the AOs Phloretin, Silymarin, Hesperetin, and Resveratrol reduced the production of both of these key proinflammatory cytokines by PBMCs is significant when considering their anti-inflammatory potential.

Also noteworthy was the inhibition of IFN-*γ* production. IFN-*γ* has long been known to prime MΦs for innate activation by LPS or to induce classical activation in conjunction with TNF-*α* [30]. In response to these signals MΦs produce a plethora of proinflammatory cytokines which include TNF-*α*, IL-1, and IL-6. Inhibition of IFN-*γ* production at sites of inflammation would be expected to reduce MΦ activation and further contribute to reduced inflammation.

The production of chemokines by activated cells is important for the influx of leukocytes to sites of local inflammation, with the specificity of these chemokines dictating the types of leukocytes being recruited. All of these AOs conferred inhibition of CCL2 production. CCL2 is the principal chemokine responsible for monocyte recruitment and can also promote CD4+ and CD8+ T cell migration [31]. Similarly all treatments resulted in a decrease in the production of CCL5, a chemokine also involved in monocyte chemotaxis. Production of the chemokines MIP-1*α* and MIP-1*β*, two structurally similar chemokines with differing signaling properties, was also reduced in the presence of the AO Silymarin. Silymarin inhibited the production of both MIP-1*α* and MIP-1*β*. Both subtypes of MIP-1 are chemotactic for a wide range of leukocytes including monocytes, T cells, MΦs, DCs, and neutrophils [32, 33]. CXCL5, a chemokine important for the chemotaxis of neutrophils, was inhibited by the AOs Phloretin, Silymarin, and Resveratrol. CXCL5 promotes neutrophil chemotaxis and also activation via an increase in intracellular calcium levels. Inhibition of the chemokines identified here, namely, CCL2, CCL5, MIP-1*α*, MIP-1*β*, and CXCL5, would likely have the greatest impact on the recruitment of neutrophils and monocytes. Given the importance of these two leukocytes during the acute and later stages of inflammation, a reduction in their recruitment should result in a reduced severity and duration of inflammation.

The inhibition mediated by AOs extended to the release of the anti-inflammatory mediators IL-1Ra and IL-10. At face value, the reduced production of these factors would be expected to promote proinflammatory responses; however, it is important to remember that these factors are also induced by innate stimuli, such as LPS, and that they self-regulate to curb excessive activation [34, 35]. Furthermore the kinetics of their expression are such that peak expression occurs after that of the proinflammatory cytokines is also induced, correlating with the transition from the acute phase of inflammation to the resolution phase.

We considered the reduction in IL-10 and IL-1Ra production alongside that of proinflammatory mediators to be suggestive of a common mechanism of inhibition, one possibility being reduced NF-*κ*B activation. To investigate the mechanism of inhibition, we measured the levels of TNF-*α* mRNA present. Silymarin and Resveratrol reduced TNF-*α* mRNA expression by approximately 50% and 75%, respectively. This observation could be explained either by a reduction in mRNA transcription due to reduced NF-*κ*B activation or by posttranscriptional mechanisms, such as the targeting of mRNA for degradation, the induction of factors which increase mRNA instability, or translational silencing. To further investigate these possibilities, we measured the mRNA expression levels of I*κ*B-*α*. Previous studies have utilized RT-PCR measurement of I*κ*B-*α* level to quantify the transcriptional power of NF-*κ*B [36]. If the inhibitory effects of the AOs occurred at the posttranscriptional level and were not due to decreased NF-*κ*B activity then levels of I*κ*B-*α* should remain unaltered. As levels of I*κ*B-*α* mRNA expression were also reduced with AO treatment, we consider it likely that the inhibitory effect of the AOs is mediated, at least in part, through reduced activation of NF-*κ*B. This would be consistent with the observed decrease in anti-inflammatory mediators (i.e., IL-10 and IL-1Ra), which are also induced by NF-*κ*B activation, and is supported by previous studies [10, 37].

It is possible that combinations of AOs would confer a greater degree of inhibition than single doses. We pretreated PBMCs with different combinations of the AOs and challenged them with LPS as described above. Significantly, for many of the inflammatory mediators assayed, the combinations were equally as effective as AOs alone despite each being present at half the concentration (Table 1, supplementary data). Notably, levels of MIP-1*β* in the presence of Phloretin plus Hesperetin were lower compared to single doses. Combinations of the AOs studied here have previously demonstrated synergistic effects in related cell types and conditions [17]. Although in the present study these data did not reach statistical significance, further investigation is indicated. Indeed, identifying single and AO combinations may provide novel therapeutic options for use in the treatment of inflammatory disorders. We also examined 2 additional AOs, Oleuropein and Ferulic Acid, in this study (Supplementary Table 1) but did not identify any significant effects on inflammatory mediators at the concentrations and combinations tested. It is possible that these AOs require significantly higher doses than what was examined and/or longer pretreatment times to see an effect.

This study has limitations including the low number of biological replicates. Larger studies with greater number of donors are required as is further research evaluating NF-*κ*B activity, IL-8, and neutrophil-specific effects. Nevertheless, our study provides evidence of the potent anti-inflammatory properties of Phloretin, Silymarin, Hesperetin, and Resveratrol on immune cells.

## 5. Conclusion

Our data further characterises the potent anti-inflammatory properties of the AOs Phloretin, Silymarin, Hesperetin, and Resveratrol. LPS-stimulated PBMCs treated with these AOs displayed an altered cytokine profile including reduced levels of the proinflammatory mediators TNF-*α*, IL-1*α*, IL-1*β*, IL-6, and IFN-*γ*. The anti-inflammatory properties of these AOs also extended to the production of several chemokines, namely, CCL2, CCL5, MIP-1*α*, MIP-1*β*, and CXCL5. Reduction in these chemokines would particularly impact the recruitment of monocytes and neutrophils to sites of local inflammation. Levels of G-CSF and GM-CSF which promote myeloid differentiation and potentiate activation were also reduced. The inhibitory effects of these AOs were present at the level of mRNA expression, most likely due to reduced NF-*κ*B activation as indicated by the reduced level of I*κ*B-*α* mRNA present. In conclusion, the AOs Phloretin, Silymarin, Hesperetin, and Resveratrol significantly inhibit the inflammatory response of PBMCs to LPS.

## Supplementary Material

Peripheral Blood Mononuclear Cells (PBMC) from 3 biological donors were activated with lipopolysaccharide from Aggregatibacter actinomycetemcomitans (Y4) and the supernatant was assayed for TNF-*α*, IL-1*α*, IL-1*β*, G-CSF, GM-CSF, IFN-*γ*, IL-6, IL-10, MCP-1, CCL5, MIP-1*α*, MIP-1*β*, IL-Ra, CXCL5, CXCL8 (IL-8), VEGF, IL-4, FGF basic, IL-2, IL-5, IL-17, and TPO after 18 hrs via cytometric bead array.
